# Risk factors of catheter – related infection in patients undergoing hemodialysis using non – tunneled double lumen catheter at Dr. Kariadi Hospital Semarang

**DOI:** 10.1017/ash.2025.92

**Published:** 2025-09-03

**Authors:** Mila Astrilia, Dwi Lestari Partiningrum, Retty Kharisma Sari, Nur Farhanah, Muchlis Achsan Udji Sofro

**Affiliations:** 1 Internal Medicine Resident, Department of Internal Medicine, Faculty of MedicineDiponegoro University; 2 Nephrology and Hypertension Division, Department of Internal Medicine, Faculty of MedicineDiponegoro University; 3 Tropical Medicine and Infectious Disease Division, Department of Internal Medicine, Faculty of MedicineDiponegoro University

**Keywords:** Catheter - Related Infection, Hemodialysis, Risk factors

## Abstract

**Introduction:** Hemodialysis (HD) is the most common renal replacement therapy modality for chronic kidney disease patients. Nearly 80% of patients starting HD use a non-tunneled double lumen (DL) catheter as the first vascular access. However, the use of this access may increase the risk of both exit site and bloodstream infections. This study aims to identify the risk factors for infection related to non-tunneled DL catheters in HD patients at Dr. Kariadi Hospital, Semarang, Indonesia. **Methods:** A retrospective cross-sectional study design was applied among adult patients who underwent HD using non-tunneled DL catheter in the Hemodialysis Unit at Dr. Kariadi Hospital between January 2022 and March 2024. Data were collected from medical histories and patients’ medical records, then analyzed using SPSS 21. P-values less than 0.05 were considered statistically significant. **Results:** This study involved 72 adult HD patients, with 58% male subject. Among them, 23 (31.9%) subjects experienced infections related to non-tunneled DL catheter. These infections included exit site infections (21%) and bloodstream infections (95%). The most dominant microorganism in infected patients was *Staphylococcus aureus*. The location of catheter insertion in the femoral vein (*p = 0.03*) and a high white blood cell count (*p = 0.03*) were significant risk factors for infection. However, factors such as age, diabetes mellitus, duration of catheter insertion > 3 months, serum iron levels, hypoalbuminemia, and anemia were not significant risk factors (*p > 0.05*). **Conclusion:** In conclusion, catheter insertion in the femoral vein and a high white blood cell count were identified as contributing factors to infections related to non-tunneled DL catheters in HD patients.

